# Shrimp injection with dsRNA targeting the microsporidian EHP polar tube protein reduces internal and external parasite amplification

**DOI:** 10.1038/s41598-024-55400-2

**Published:** 2024-02-28

**Authors:** Satika Yuanlae, Tharinthon Prasartset, Onrapak Reamtong, Natthinee Munkongwongsiri, Muthita Panphloi, Thanchanok Preechakul, Rungkarn Suebsing, Siripong Thitamadee, Anuphap Prachumwat, Ornchuma Itsathitphaisarn, Suparat Taengchaiyaphum, Chanadda Kasamechotchung

**Affiliations:** 1 Department of Biochemistry, Faculty of Science, Mahidol University, Rama VI Rd., Bangkok, 10400 Thailand; 2 Center for Excellence in Shrimp Molecular Biology and Biotechnology (Centex Shrimp), Faculty of Science, Mahidol University, Rama VI Rd., Bangkok, 10400 Thailand; 3 Department of Biotechnology, Faculty of Science, Mahidol University, Rama VI Rd., Bangkok, 10400 Thailand; 4 Department of Molecular Tropical Medicine and Genetics, Faculty of Tropical Medicine, Mahidol University, Bangkok, 10400 Thailand; 5 Aquatic Animal Health Research Team (AQHT), Integrative Aquaculture Biotechnology Research Group, National Center for Genetic Engineering and Biotechnology (BIOTEC), National Science and Technology Development Agency (NSTDA), Yothi Office, Rama VI Rd., Bangkok, 10400 Thailand; 6 Enzyme Technology Laboratory, National Center for Genetic Engineering and Biotechnology (BIOTEC), 113 Thailand Science Park, Phaholyothin Road, Khlong Luang, Pathumthani, 12120 Thailand; 7 Analytical Sciences and National Doping Test Institute, Mahidol University, Rama VI Rd., Bangkok, 10400 Thailand; 8 Department of Fisheries, Faculty of Agriculture and Natural Resources, Rajamangala University of Technology Tawan-ok, Chonburi, 20110 Thailand

**Keywords:** *Enterocytozoon hepatopenaei* (EHP), Germination process, Polar tube protein 2 (EhPTP2), *Penaeus vannamei*, EhPTP2-dsRNA, Biotechnology, RNAi therapy

## Abstract

The microsporidian *Enterocytozoon hepatopenaei* (EHP) is a major threat to shrimp health worldwide. Severe EHP infections in shrimp cause growth retardation and increase susceptibility to opportunistic infections. EHP produces spores with a chitin wall that enables them to survive prolonged environmental exposure. Previous studies showed that polar tube extrusion is a prerequisite for EHP infection, such that inhibiting extrusion should prevent infection. Using a proteomic approach, polar tube protein 2 of EHP (EhPTP2) was found abundantly in protein extracts obtained from extruded spores. Using an immunofluorescent antibody against EhPTP2 for immunohistochemistry, extruded spores were found in the shrimp hepatopancreas (HP) and intestine, but not in the stomach. We hypothesized that presence of EhPTP2 might be required for successful EHP spore extrusion. To test this hypothesis, we injected EhPTP2-specific double-stranded RNA (dsRNA) and found that it significantly diminished EHP copy numbers in infected shrimp. This indicated reduced amplification of EHP-infected cells in the HP by spores released from previously infected cells. In addition, injection of the dsRNA into EHP-infected shrimp prior to their use in cohabitation with naïve shrimp significantly (p < 0.05) reduced the rate of EHP transmission to naïve shrimp. The results revealed that EhPTP2 plays a crucial role in the life cycle of EHP and that dsRNA targeting EHP mRNA can effectively reach the parasite developing in host cells. This approach is a model for future investigations to identify critical genes for EHP survival and spread as potential targets for preventative and therapeutic measures in shrimp.

## Introduction

Microsporidia are obligate intracellular parasites with a characteristic infectious spore that contains an invasion organelle called a polar tube. A double-layered spore wall, consisting of an outer layer (exospore) and a chitin-rich inner layer (endospore), allows the spore to withstand harsh external environments^1^. Upon induction or under opportune conditions, the spore extrudes its polar tube to pierce a host cell in order to initiate an intracellular life cycle in a process called germination^2^. The polar tube serves as a conduit through which sporoplasm is transferred into the host cell. Many stimuli have been reported to trigger spore germination as reviewed by Weiss and colleagues^3^.

The polar tube is a supramolecular complex of different polar tube proteins. To date, seven polar tube proteins have been identified (PTP1–PTP7)^3–8^. Among them, PTP2 has been previously studied in various microsporidian species. The homologs share a myriad of properties, including a lysine rich sequence, a signal peptide at the N-terminus, a basic isoelectric point (pI), and conserved cysteines^4,7,9^.

*Enterocytozoon hepatopanaei* (EHP) is a microsporidian that infects the hepatopancreas (HP) and intestine of penaeid shrimps including *Penaeus vannamei* and *P. monodon*^10,11^. The transmission of EHP occurs horizontally through cannibalism and cohabitation^11,12^. Despite having little significant effect on shrimp mortality, EHP infection increases the feed consumption ratio (FCR) and may decrease average daily growth (ADG)^13^. Furthermore, an EHP load greater than 10^3^ copies per nanogram (ng) of total hepatopancreatic DNA negatively correlates with shrimp growth^14^. Thus, a severe infection in shrimp leads to a retarded growth and a subsequent reduction in productivity^15,16^.

Heat inactivation of EHP spores has been demonstrated to prevent spore extrusion, which consequently inhibits the infectivity of the spore, suggesting that the spore extrusion process is prerequisite for EHP infection in shrimp^17^. It has been shown that polar tube protein 2 (PTP2) of EHP (EhPTP2) is a 284-amino acid protein that shares common characteristics with its homolog in other microsporidians^7^. Furthermore, an immunofluorescence assay (IFA) using an antibody against EhPTP2 indicated that EhPTP2 is localized on the polar tube of germinated spores, suggesting a new strategy for diagnosing EHP infection^7^. However, further studies of EhPTP2 on its role in pathogenesis during EHP infection are still needed.

RNA interference (RNAi) is a post-translational gene silencing mechanism that is mediated by a sequence-specific double-stranded RNA (dsRNA). The technique has been applied in various studies of shrimp viruses in both preventative and therapeutic settings. As a therapeutic tool, an injection of dsRNA targeting the protease of Yellow Head Virus (YHV) into YHV-infected shrimp, for instance, results in a reduction in viral load and mortality^18^. In addition, it has been shown that a preventative injection of dsRNA specific to structural or non-structural ORFs of *Penaeus stylirostris* densovirus (*Pst*DNV) into naïve shrimp prior to a viral challenge inhibited *Pst*DNV replication^19^. Similarly, knockdown of PTP3 of *Nosema ceranae* (a microsporidian infecting the honeybee *Apis mellifera*) by a microsporidian specific dsRNA led to a reduction in spore load in infected animals^20^. Therefore, these previous findings support the hypothesis that the silencing of PTPs by RNAi would reduce microsporidian infection.

In this study, we hypothesized that EhPTP2, is associated with spore germination. Therefore, we predicted that silencing of EhPTP2 would inhibit EHP replication, reduce EHP copy numbers, and reduce EHP transmission. To begin, a proteomic approach was used to identify proteins involved in the germination process in the white leg shrimp, *Penaeus vannamei*. Then, the extrusion sites for EHP spores along the shrimp digestive tract and the effects of EhPTP2 dsRNA in suppressing EHP infection were investigated. This research provides a platform for further development of possible therapeutic or preventive treatments for EHP infection based on the suppression of EhPTP2.

## Materials and methods

### Proteomic approach to identify proteins involved in EHP spore germination

To identify proteins involved in the spore germination process, EHP spores were isolated from the hepatopancreas (HP) of EHP-infected shrimp (*P. vannamei*) according to previously described methods^21^. The purified spores were incubated with 2% Phloxine B for 20 min and then sequentially washed with sterile water. The germinated spore pellet was further mixed with a protein lysis buffer (8 M Urea, 2 M Thiourea, 2% CHAPS, 50 mM Dithiothreitol (DTT), 1× protease inhibitor mix) and vortexed for 5 min before centrifugation at 12,000 rpm for 5 min to collect soluble protein. The total protein concentration was determined by the Bradford’s assay using bovine serum albumin (Bio-Rad, USA) as a standard protein. The quantity of 30 μg of the EHP spore proteins were separated by a 12% SDS-PAGE. The gel was stained with Coomassie Brilliant Blue G-250 (CBB G-250) overnight and destained with 10% acetic acid. The protein bands obtained by SDS-PAGE were sliced and subjected to in-gel trypsin digestion. To remove the stained dye, small pieces of gel (1 mm^3^) were immersed in 50% methanol for 1 h. The clear gel piece was then dehydrated with 100% acetonitrile and incubated with 10 ng/μl sequencing-grade porcine trypsin (Promega, USA) containing a digestion buffer (25 mM ammonium bicarbonate, pH 8.5) at 37 °C for 18 h. The digested peptides were then extracted twice with 50% acetonitrile in the digestion buffer. The resulting peptide solution was pooled in a new tube and then lyophilized by a vacuum pump. The tube was kept at − 80 °C until subject to mass spectrometry. Peptide identification was identified by using Nano-liquid chromatography tandem mass spectrometry (Bruker Daltonics, USA). The most abundant EHP spore proteins were selected according to the Exponentially Modified Protein Abundance Index (emPAI) value which quantifies the exponential relationship between molar protein concentration and the Protein Abundance Index (PAI). The emPAI is calculated as emPAI = 10^PAI^, where PAI represents the ratio of observed peptides to observable peptides.

### Polar tube protein 2 of EHP (EhPTP2) recombinant protein synthesis and polyclonal antibody production

A recombinant plasmid pGEX-4 T-1 harboring a full-length EhPTP2 gene (GenBank accession no. OQS55341.1) was synthesized by Genscript, Singapore. The recombinant plasmid was transformed to the *Escherichia coli* strain BL-21(DE) for recombinant protein synthesis. After bacterial induction by 1 mM IPTG (Isopropyl ß-D-1-thiogalactopyranoside) at 37 °C for 4 h, the bacterial cell was harvested by centrifugation. Subsequently, the recombinant protein was purified by using Glutathione Sepharose 4B beads (GE Healthcare, USA). Purified protein was analyzed on 12.5% SDS-PAGE with CBB G-250 staining.

Polyclonal antibody production was performed by China Peptides Co., Ltd. In brief, 0.5 mg of the purified EhPTP2 protein was injected into a rabbit two times during a 7-day interval. After the second round of immunization, rabbit serum was collected, and IgG was purified using a protein A/G affinity column. The titers of the purified IgG were tested by the ELISA method against the purified recombinant EhPTP2. Testing of the antibody specificity to the protein target was performed using an EHP spore extract. The total protein of EHP spores was extracted under two conditions as either whole spore protein extract or partially purified polar tube protein extract. For the whole spore protein extract, the purified spores were mixed with protein lysis buffer and an equal volume of 0.5 µm-glass beads (Sigma, USA) before homogenization with a bead beater for 5 min. The supernatant was collected by centrifugation. For polar tube extraction, the spores were purified and incubated with 2% Phloxine B prior to protein extraction as mentioned earlier. The total protein concentration was determined by Bradford’s assay.

Twenty micrograms of spore protein were resolved on 12.5% SDS-PAGE and transferred to a PVDF membrane using a semi-dry blotting system (Bio-Rad, USA). The membrane was further incubated with a blocking solution (5% skimmed milk in 1× Tris-buffered saline (TBS) pH 7.6 with 0.5% Tween®-20) for 1 h at room temperature (RT). The membrane was further incubated with the rabbit anti-EhPTP2 primary antibody (1:2000) overnight at 4 °C. After washing the membrane three times for 10 min each with a wash buffer (1× TBS, pH 7.6, with 0.5% Tween®-20), the membrane was incubated with 1:2500 goat anti-rabbit alkaline phosphatase conjugated secondary antibody (Sigma, USA) for 1 h at RT. After membrane washing, the immune signal was developed using NBT/BCIP substrate (Roche, Germany). The SDS-PAGE stained gel and blotting membrane were photographed and analyzed by ImageLab software (Bio-Rad, USA) version 6.0 (https://www.bio-rad.com/ImageLab).

### Immunofluorescence analysis of EhPTP2 in EHP spores and detection of spore accumulation in a shrimp digestive tract

To determine EHP spore extrusion sites in the shrimp digestive tract, 20 naturally EHP-infected shrimp (*P. vannamei*, 3–5 g body weight) obtained from a shrimp farm were dissected to collect the stomach, HP and the intestine from each. Each tissue was separately pooled in individual 15-ml sterile conical tubes. The pooled tissues were homogenized and the EHP spores were purified following a previously reported protocol^21^. Spores collected from each Percoll (GE Healthcare, USA) layer (25%, 50%, 75%, and 100%) were dropped on a circle coverslip and fixed with a 4% paraformaldehyde (PFA) solution at 4 °C for 18–20 h. After the fixation, the spores were washed with phosphate-buffered saline (PBS) (pH 7.4) and stored in PBS at 4 °C. The extruded spores were identified by an immunofluorescence assay (IFA). The fixed spores were washed with 1× PBST (1× PBS with 0.5% Tween® 20) and incubated with 10% fetal bovine serum (FBS) for 1 h at RT to block non-specific interactions. The anti-EhPTP2 antibody was used as a primary antibody (1:500 dilution in 1% FBS) and incubated at 4 °C for 18 h. After that, the anti-EhPTP2 antibody was removed and washed twice with PBS. An AlexaFluor® conjugated antibody (GAR-Alexa 555, Invitrogen, USA) was used as a secondary antibody (1:500 dilution in 1% FBS) and incubated for 1 h at RT in the dark. To detect the chitin wall of EHP, the spores were stained with a calcofluor white dye (Sigma, USA) followed by ProLong™ Gold Antifade Mountant mix with DAPI (4′,6-diamidino-2-phenylindole) (Invitrogen, USA). The stained spores were observed under the Zeiss LSM 800 confocal laser scanning microscope.

### An immunohistochemical assay to monitor EHP localization in shrimp

To obtain EHP-infected shrimp, a laboratory cohabitation assay was set up according to a previous study^12^. Thirty naïve shrimp were co-cultured with 15 EHP-infected shrimp, which were placed in a plastic cage. The naïve shrimp were randomly collected on days 1, 3, 5 and 7 after the cohabitation. To monitor EHP localization in the shrimp digestive tract, immunohistochemistry was performed using an antibody specific to EhPTP2. At designated time-points, shrimp were randomly collected and the whole part of shrimp digestive tract was dissected and fixed with 4% PFA for 24 h prior to tissue processing. The tissues were sectioned and placed on a positively charged glass slide. Then, the slide was dried on a hot plate at 60 °C for 1 h, deparaffinized with xylene, and rehydrated with absolute ethanol. To suppress endogenous peroxidase activity and to reduce background signals, the section was incubated in 3% hydrogen peroxide (H_2_O_2_) in 70% ethanol for 30 min and in 1% glycine in PBS for 30 min at RT. Antigen retrieval was performed by incubating the section in a boiled 1× citrate buffer for 10 min and washing the section with a 1× PBST buffer for 5 min followed by a final wash in a 1× PBS buffer with 0.4% triton X-100 for 5 min. To reduce nonspecific interactions, the section was incubated in a blocking solution (10% FBS in 1× PBS, pH 7. 4) for 1 h and washed with 1× PBST twice. Then, the section was incubated in a primary antibody solution (1:500 dilution of a rabbit anti-EhPTP2 antibody, raising from the recombinant EhPTP2 protein, in 10% FBS, 1× PBS, pH 7.4) overnight at 4 °C. Subsequently, the section was washed with 1× PBST twice and incubated in 1:500 goat anti-rabbit secondary antibody diluted in 10% FBS, 1× PBS, pH 7.4 for 2 h, at RT. Finally, the slide was washed several times with 1× PBST and incubated in a detection buffer for an NBT/BCIP substrate (Roche, Germany). The signal development was stopped by washing with Tris–EDTA (TE) buffer. Then, the section was counterstained with Bismarck brown (Sigma, USA). The stained slide was dried by immersing in xylene prior to mounting the slide by using a mounting solution. The immunostained slide was examined under a light microscope.

### The effect of EhPTP2-specific double-stranded RNA (dsRNA) on EHP replication and transmission in shrimp

To evaluate the role of EhPTP2 in EHP replication and transmission, suppression of EhPTP2 gene by dsRNA in both naïve and EHP-infected shrimp was performed. In vitro synthesis of dsRNA was carried out by using a large-scale dsRNA production kit (Promega, USA). The candidate region for a long dsRNA against the EhPTP2 gene was designed by the *E-RNAi* web service (http://www.e-rnai.org/). The target dsRNA region was amplified by single step PCR and then the PCR amplicon was ligated into a pGEM-T easy vector (Promega, USA) to generate a DNA template (see primer list in supplementary information, Table S1). Two PCR templates containing a DNA fragment encoding the EhPTP2dsRNA with flanking T7 promoters orienting in opposite directions were amplified by a single step PCR and purified using a Gel/PCR purification kit (GeneAids, Taiwan). Sense- and anti-sense strands were synthesized separately by in vitro transcription following the manufacturer’s protocol. The dsRNA was purified by phenol–chloroform-isopropanol precipitation. A EhPTP2-dsRNA pellet was dissolved in RNase-free sterile water prior to determining RNA concentration by a Nanodrop spectrophotometer (ThermoFisher Scientific, USA).

To determine the effect of EhPTP2-dsRNA on EHP replication, EHP-infected shrimp were intramuscularly (between 3rd and 4th abdominal segments) injected with 10 µg EhPTP2-dsRNA per gram body weight. Enhanced green fluorescent protein (EGFP) dsRNA was also synthesized and injected as a non-specific dsRNA. The dsRNA was injected every 7 days during a period of 21 days. For a negative control, shrimp were injected with 1× PBS, pH7.4. To determine the effect of the dsRNA injection, 4 shrimp were randomly collected every 7 days for HP dissection and subjected to nucleic acid extraction (see diagram in Fig. 4A). The reduction of EhPTP2 transcript expression was determined by RT-PCR using SuperScript™ III One-Step RT-PCR System with Platinum™ *Taq* DNA Polymerase (Invitrogen, USA), and the EHP replication levels in shrimp were determined by a one-step PCR using One*Taq*® 2X Master Mix with Standard Buffer (New England Biolabs, USA) using the SWP primers^22^.

To determine the effects of EhPTP2-dsRNA on EHP transmission to EHP-free shrimp, 10 EHP-infected shrimp were injected with EhPTP2-dsRNA (or EGFP-dsRNA) as described in the previous experiment prior to co-culturing with naïve shrimp. Three doses of the dsRNA were injected three times (on day 1, 7 and 14). Seven days after the third injection, 10 naïve shrimp were cohabitated with the injected subjects and maintained for 14 more days. The HP were randomly collected from the co-cultured shrimp for nucleic acid extraction (see diagram in Fig. 5A). The EhPTP2 transcript level was determined by RT-PCR and the EHP replication level was determined in the same shrimp by a single step PCR. The PCR amplicons were separated 1.5% agarose gel electrophoresis and staining by ethidium bromide. The agarose gel image was captured by the Gel Doc™ EZ Gel Documentation System (Bio-Rad, USA) and image analyses by ImageLab software (Bio-Rad, USA) version 6.0 (https://www.bio-rad.com/ ImageLab). The representative gel area was processed and rearranged by Microsoft PowerPoint version 2016.

### Statistical analysis

The relative expression level of EHP was calculated based on intensities of EHP amplicons compared to that of an internal control gene (actin). The amplicon intensities were determined using ImageLab analysis software (Bio-Rad, USA) version 6.0 (https://www.bio-rad.com/ ImageLab). Multiple comparisons of EHP replication were analyzed by One-Way ANOVA (p < 0.05). The bar graphs and multiple comparison tests were generated using GraphPad Prism version 7.0.

### Ethic statement

This work followed Thailand’s laws for ethical animal care under the Animal for Scientific 83 Purposes ACT, B.E. 2558 (A.D. 2015) under project approval number BT-Animal document no. BT-Animal 18/2565) from the National Center for Genetic Engineering and Biotechnology (BIOTEC), National Science and Technology Development Agency (NSTDA), Thailand.

## Results

### EHP polar tube protein 2 (EhPTP2) detection in protein extracts from germinated spores

Following the confirmation that EHP spore extrusion was essential for EHP infection in shrimp^17^, a proteomic analysis of extruded spores was performed to pinpoint the critical proteins responsible for the spore extrusion process. From the extruded spore extract, approximately 450 proteins were identified (see BlastP results in supplementary file 1). They could be classified into 2 groups as putative proteins (277) and hypothetical proteins (173). Among the most abundant proteins listed in Table 1, our analysis revealed the presence of polar tube protein 2 (EhPTP2) and polar tube protein 3 (EhPTP3), with respective rankings of 8 and 234. Due to its notably higher emPAI score, EhPTP2 was selected for further analysis.

**Table 1:** Most abundant EHP spore proteins obtained using the proteomic approach.

Rank	Protein	emPAI
1	Hypothetical protein EHP00_1800	7.325
2	molecular chaperone	5.745
3	Actin-1	4.955
4	Hypothetical protein EHP00_2548	4.600
5	Protein translation elongation factor EF-1A	4.525
6	Hypothetical protein EHP00_1603	4.450
7	Nucleoside	4.410
8	Polar tube protein 2	4.075
9	RPS27D	4.070
10	rps21	3.545
234	PTP3	0.170

### The hepatopancreas and intestine are the initial extrusion sites of EHP spores

Our study found that the EhPTP2 protein is expressed in proteins extracted from both total spore and polar tube portions. The apparent molecular weight of 37 kDa (Supplementary Fig. S1) suggested that there may by post-translational modifications such as O-glycosylation^7^. A previous study illustrated that germinated spores can be detected by an immunofluorescence technique using an antibody against the EhPTP2 protein^7^. Along with the previous study, we confirmed that PTT2 protein was localized along extruded polar tubes (Supplementary Fig. S2).

**Figure 1:**
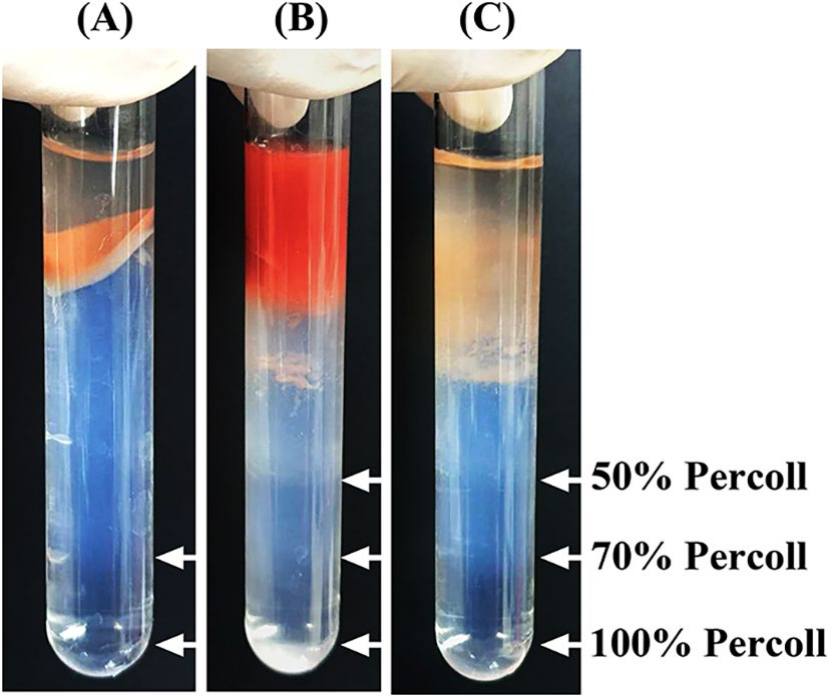
The digestive tract extracts of shrimp fractionated by Percoll gradient centrifugation. (**A**) stomach, (**B**) hepatopancreas, and (**C**) intestine. White arrows indicate EHP spore-containing bands after centrifugation.

**Figure 2:**
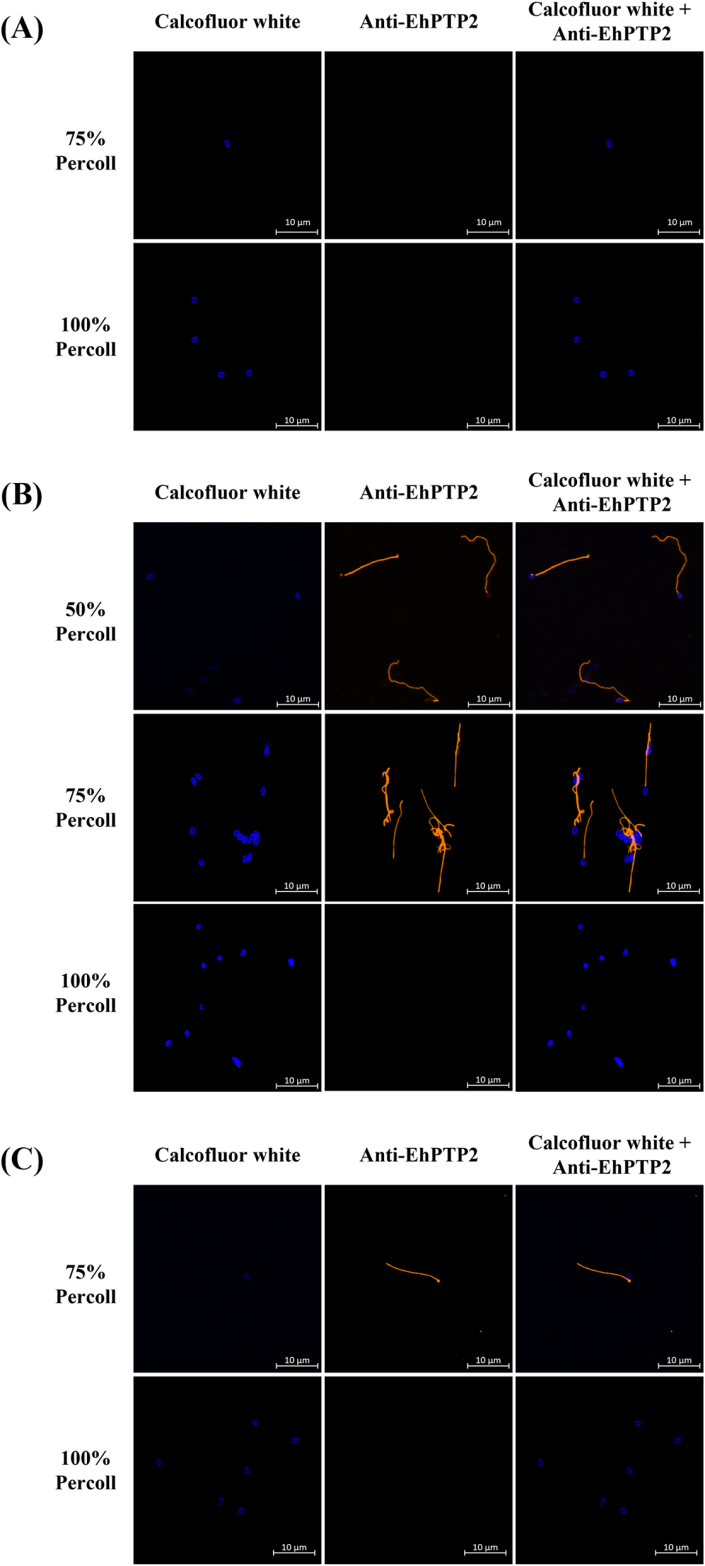
Photomicrographs of germinated EHP spores in the hepatopancreas and intestine. Immunofluorescence assays detecting germinated EHP spores (orange fluorescence from anti-EhPTP2 and Alexa Fluor® conjugated antibodies, GAR-Alexa 555) in 50%, 75%, and 100% Percoll layers isolated from (**A**) stomach, (**B**) hepatopancreas, and (**C**) intestine samples. The EHP spores show blue fluorescence from staining with Calcofluor White stain.

**Figure 3:**
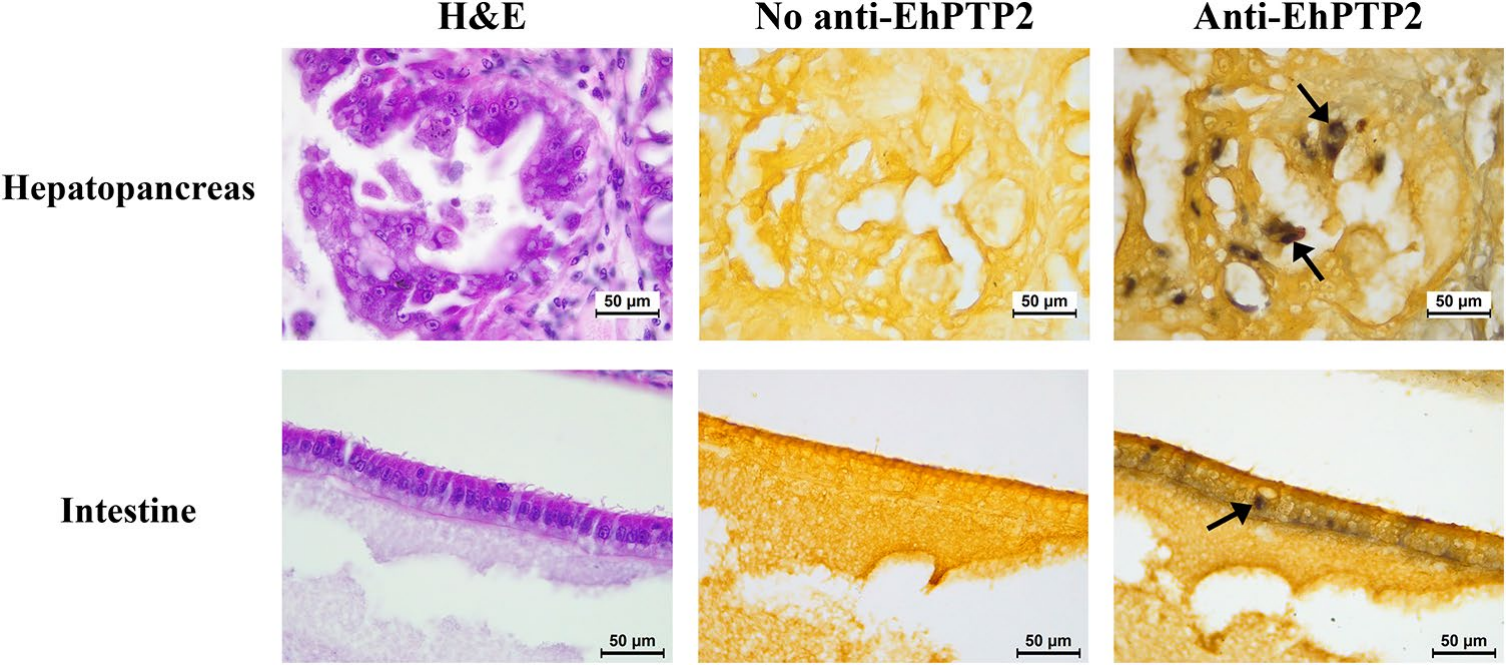
EhPTP2 detected in the hepatopancreas and intestine using immunohistochemistry. Photomicrographs of 3 consecutive sections of shrimp tissue from a sample collected after 7 days of cohabitation were analyzed by Hematoxylin and Eosin (H&E) staining (left column), negative control immunohistochemistry (middle column), and immunohistochemistry for detecting EhPTP2 using an anti-EhPTP2 antibody (right column). Dark brown precipitates indicating positive signals for EhPTP2 in the hepatopancreatic cells and epithelial cells of the intestine (black arrows).

To determine spore germination sites other than the hepatopancreas (HP) in the shrimp digestive tract, crude EHP spores were isolated from the stomach, HP, and the intestine of EHP-infected shrimp by Percoll gradient centrifugation. The result showed that EHP spores could be observed in 50%, 75% and 100% Percoll fractions (Fig. 1). Each fraction from the gradient was then tested for the presence of EhPTP2 by IFA. The results revealed that both germinated and non-germinated EHP spores could be detected in the HP and the intestine, whereas only non-germinated spores were present in the stomach (Fig. 2). Moreover, a time-course analysis of EHP infection in shrimp monitored by immunohistochemistry detecting EhPTP2 illustrated that EhPTP2 proteins could be observed mainly in hepatopancreatic cells but also in epithelial cells of the intestine in shrimp after 7 days of cohabitation (Fig. 3). EHP infection in both sites was also confirmed in the same samples by an in situ hybridization technique^23^ (Supplementary Fig. S3). In contrast, no signals were detected in shrimp samples collected from days 1 to 5 (Data not shown). These results indicated that spore germination happened inside the HP and intestine.

### The suppression of EhPTP2 expression by a specific dsRNA resulted in a reduction in EHP infection

A previous study found that in vivo polar tube germination was a prerequisite for the initiation of.

EHP infection^17^, and we further found that the process occurred in shrimp HP and intestine. The EhPTP2 protein was also found to be abundant in a polar tube portion which may participate in the polar tube extrusion process. Thus, we hypothesized that interruption of polar tube protein synthesis by suppressing EhPTP2 mRNAs should reduce EHP infection.

**Figure 4:**
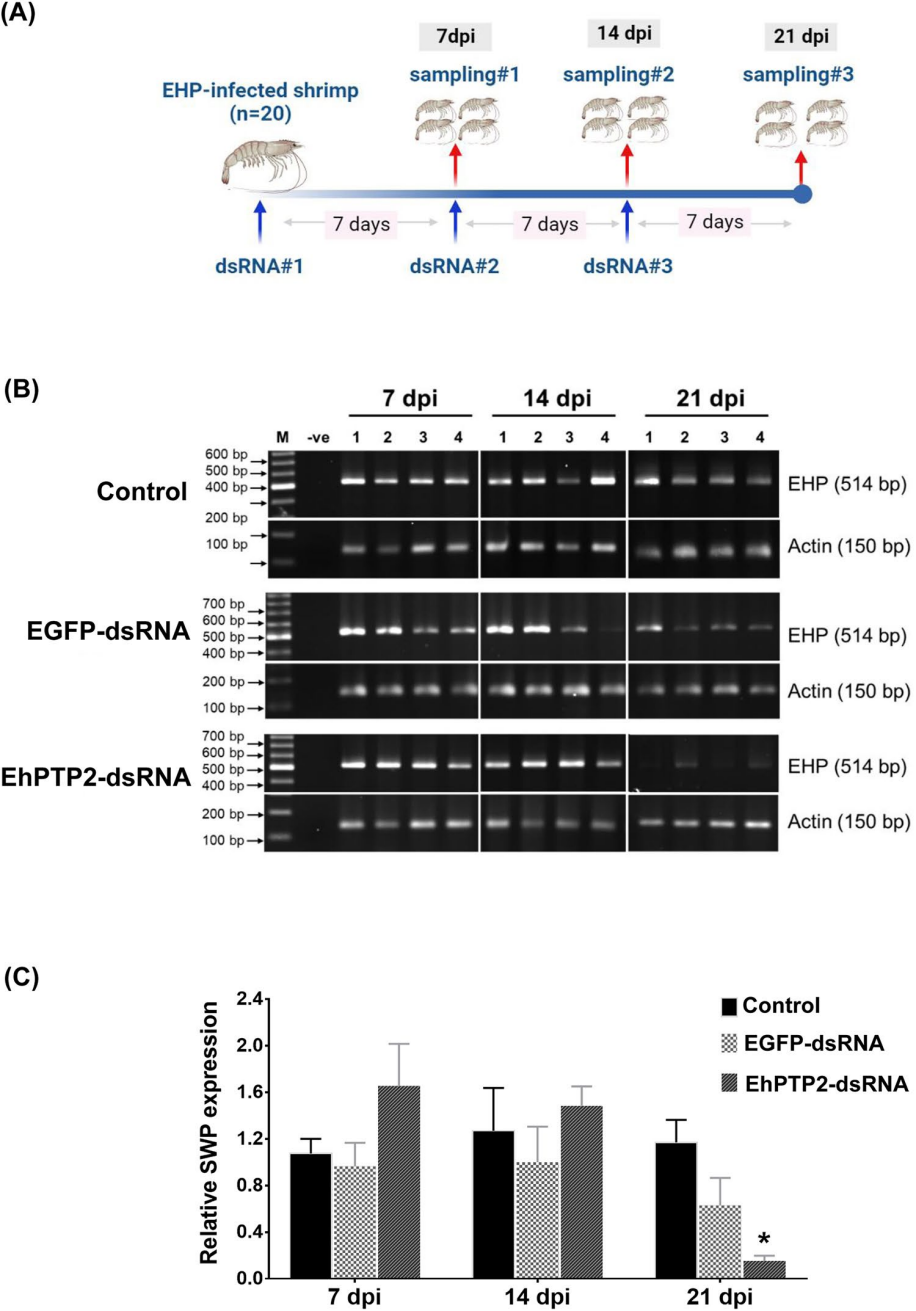
Suppression of EhPTP2 mRNA expression in EHP-infected shrimp reduced EHP replication. (**A**) A schematic diagram representing an experimental design to determine the effect of dsRNA on EHP replication. The EHP-infected shrimp were injected with three doses with EhPTP2-dsRNA at three 7-day intervals before the EHP replication level was determined. (**B**) PCR detection of SWP gene in EHP-infected shrimp after injection of PBS (control), EGFP-dsRNA or EhPTP2-dsRNA showed decreased EHP replication on day 21 post-injection with EhPTP2-dsRNA. (**C**) A bar chart showing relative SWP expression in EHP-infected shrimp at each time point after dsRNA injection revealed a significantly reduced EHP replication level (asterisk) when compared to control and non-specific dsRNA injection. Actin was used as an internal control. dpi = days post injection, M = DNA marker, − ve = negative control. *p < 0.05. The original gels are presented in Supplementary information, Figure S4–S6. The representative gel areas corresponding to this figure are labeled in red boxes. All agarose gel pictures were captured and analyzed by ImageLab software version 6.0 (https://www.bio-rad.com/ImageLab).

**Figure 5:**
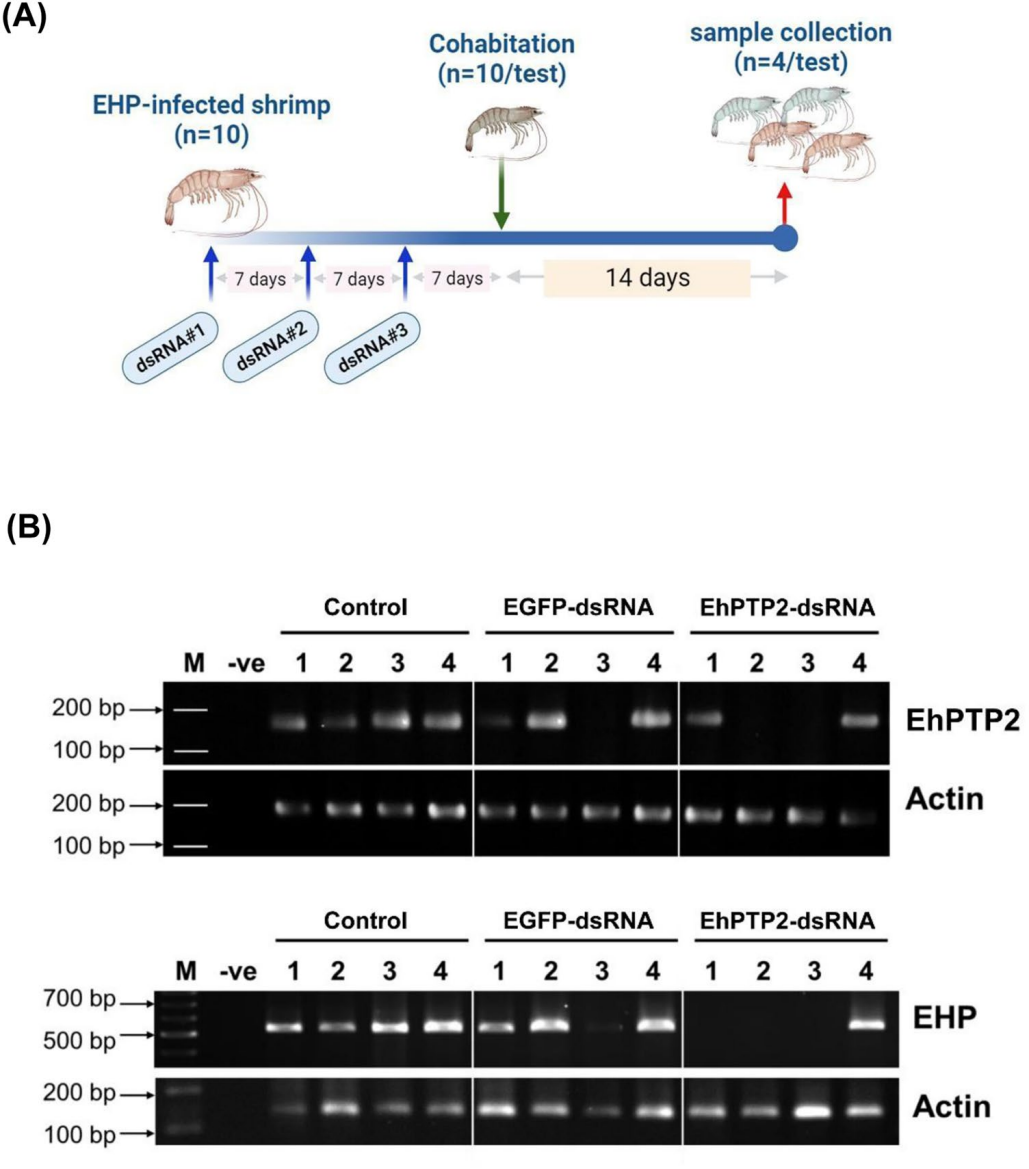
Suppression of EhPTP2 mRNA expression in EHP-infected shrimp negatively affect the horizontal transmission rate. (**A**) A diagram representing the experimental design to test how EhPTP2dsRNA affected horizontal EHP transmission to naïve shrimp. The EHP-infected shrimp were injected with three doses of dsRNA, at three 7-day intervals, and then the naïve shrimp were cohabitated for 14 days. (**B**, upper panel) RT-PCR detection of EhPTP2 transcript expression in naïve shrimp post cohabitation and PCR detection of SWP gene accumulation also indicated a low replication rate of EHP in naïve shrimp when cohabitated with EHP-infected shrimp that were previously injected with EhPTP2dsRNA (lower). Actin was used as an internal control. M = DNA marker, − ve = negative control. The original gels are presented in Supplementary information, Figure S7–S8. The representative gel areas corresponding to this figure are labeled in red boxes. All agarose gel pictures were captured and analyzed by ImageLab software version 6.0 (https://www.bio-rad.com/ImageLab).

After 21 days, EHP replication in the HP was determined by PCR. The results showed that reduction in EHP replication in infected shrimp was significantly observed by day 21 post administration of EhPTP2-dsRNA when compared to non-specific dsRNA or buffer injection (Fig. 4B,C). To determine reduction of EhPTP2 expression could affect EHP transmission, repeat experiment of EhPTP2-dsRNA administration in EHP-infected shrimp was performed as mentioned previously. After 14 day of cohabitation, detection of EHP replication in the previously EHP-free animals showed that only 25% of the naïve shrimp were infected with EHP. In contrast, 100% of the naïve shrimp reared with the control group without dsRNA administration were infected (Fig. 5B). This result indicated that the horizontal transmission of EHP could be diminished by EhPTP2 suppression.

The suppression of EhPTP2 expression in EHP-infected shrimp negatively affected the EHP life cycle in the host and, ultimately, led to a reduction in EHP infection and horizontal transmission to other shrimp. These findings supported the contention that EhPTP2 is involved in polar tube germination.

## Discussion

### IFA and IHC methods that detect EhPTP2 protein expression can be used to monitor EHP spore germination and localization in infected shrimp

EHP spore detection through light microscopy is challenging due to the requirement for a high magnification lens (100x). Targeting specific genes or proteins associated with EHP can offer more sensitive and specific detection. The polar tube protein 2 of EHP (EhPTP2) serves as a valuable target for detection due to its involvement in the polar tube extrusion process, which constitutes a critical step in the parasite's life cycle. Our research indicates that Immunofluorescence Assay (IFA) targeting the EhPTP2 protein can be employed to detect extruded EHP spores in isolated spore samples (Fig. 2; Supplementary Fig. S2). This finding suggests that IFA could be beneficial for monitoring EHP spore germination. Furthermore, beyond the detection of live spores, EhPTP2 can also be utilized as a target for detecting EHP infection in shrimp histological sections using Immunohistochemistry (IHC) (Fig. 3). Our finding supports the use of EhPTP2 detection as a diagnostic method for EHP infection, which is consistent with previous reports of EhPTP2 detection using a recombinase polymerase amplification (RPA) and CRISPR-Cas 12a fluorescence assay^24^, and an integrated qPCR and staining microscopy method^25^.

### Germination in the intestine may not lead to a fruitful infection

In addition to germinating in the HP as in previous reports^7,11^, a small number of germinated spores were isolated from the intestine purified on the Percoll gradient (Figs. 1 and 2) and detected by immunohistochemistry of EhPTP2 (Fig. 3). Along the digestive tract of shrimp, there is a gastric sieve (GS) which serves as a size-exclusion filter for particles of approximately 0.2–0.7 µm in diameter^26^. Larger particles are diverted to the intestine instead of the HP. Since the average size of EHP oval spores is approximately 1 µm long (1.1 ± 0.2 µm × 0.7 ± 0.1 µm)^10^, it is possible that the germinated spores detected in the intestine were derived from those that were size-excluded from the HP by the GS. While germinated spores and positive signals of EhPTP2 from immunohistochemistry were detected in the intestine, the evidence remains insufficient to conclude that the intestine is another proliferation site of EHP. Instead, we speculate that the presence of the germinated spores in the histology result was the remnant of fecal contamination rather than an active infection.

### Therapeutic and preventative applications of EhPTP2 knockdown

As EhPTP2 is associated with polar tube extrusion, it suggests that suppression of EhPTP2 mRNA would inhibit the extrusion process and result in reduced EHP infection. This was indeed the case when EhPTP2-specific dsRNA was intramuscularly injected into previously EHP-infected shrimp (Figs. 4 and 5). The inhibition of infection when the RNAi technique is applied preventatively is not uncommon when key virulent genes are knocked down in naïve shrimp prior to pathogen exposure^19,27–33^. In one cultured pond, it was observed high variation of EHP infection levels in the population. The 25% of shrimp injected with dsRNA and showed EHP infection might be those infected with high levels of EHP. If the fourth dose of dsRNA-EhPTP2 can be applied, it might be able to reduce the infection. What was promising, in this study, is the fact that administration of EhPTP2-specific dsRNA could be utilized therapeutically once infection had already been established in two aspects: (1) reducing EHP loads in infected shrimp and (2) reducing further EHP spread in shrimp ponds. Only a handful of reports have documented successful therapeutic applications in pathogen-infected shrimp^18,19,34,35^. Considering that EHP infection is chronic and that the negative impact on growth is not discernable until the EHP copy number reaches a critical threshold of 10^3^ copies per ng of total HP DNA^14^, it suggests that combination of routine surveillance and therapeutic application of dsRNA against EhPTP2 may have potential for controlling EHP.

### Supplementary Information

Supplementary Information 1.

Supplementary Information 2.

## Data Availability

The datasets generated and analysed during the current study are available in the Supplementary file 1 for protein identification by Mass spectrometry and Supplementary information, respectively. Additional data requirement, please contact: Dr. C. Kasamechotchung (chanadda_ka@rmutto.ac.th) or Dr. S. Taengchaiyaphum (suparat.tae@biotec.or.th).
