# Supplementary material for: Shrimp injection with dsRNA targeting the microsporidian EHP polar tube protein reduces internal and external parasite amplification
Source: Sci Rep. 2024 Feb 28;14:4830. doi: 10.1038/s41598-024-55400-2 (PMC10899260; doi:10.1038/s41598-024-55400-2)
Supplement: Supplementary file 2 — Supplementary Information 2. [file 41598_2024_55400_MOESM2_ESM.docx]

**Supplementary information**


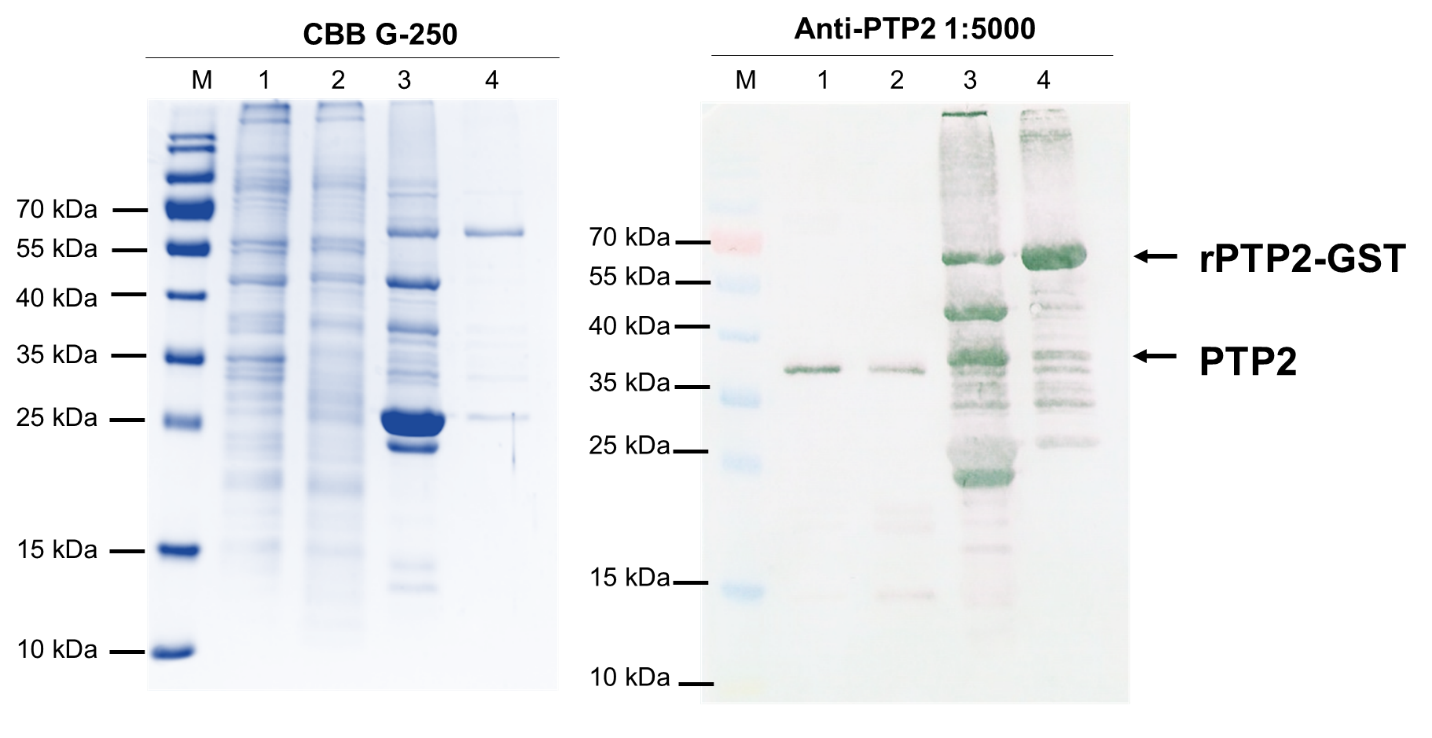


**Figure S1. Validation of the anti-EhPTP2 polyclonal antibody by Western blot analysis.** The protein extracts of polar tubes and whole EHP spores were separated. The presence of EhPTP2 was determined by Western blot analysis. The **Left panel** is SDS-PAGE stained with CBB-G250 and shows (Lane 1) the protein extract derived from whole spore; and (Lane 2) the polar tube fraction, the purified EhPTP2 recombinant protein tagged with GST (rEhPTP2-GST) and (Lane 3) with protease cleavage of GST tag or (Lane 4) without cleavage, which were used as the positive controls for antibody detection; (Lane 3) The **Right panel** is the Western blot of the same gel for the EhPTP2 protein of approximately 37 kDa showing (Lane 1) the whole EHP spore protein extract and (Lane 2) the polar tube portion. Note that the upper part of stacking gel was removed prior to apply for CBB-G250 staining and semi-dry blot on membrane, and then followed by antibody incubation process.


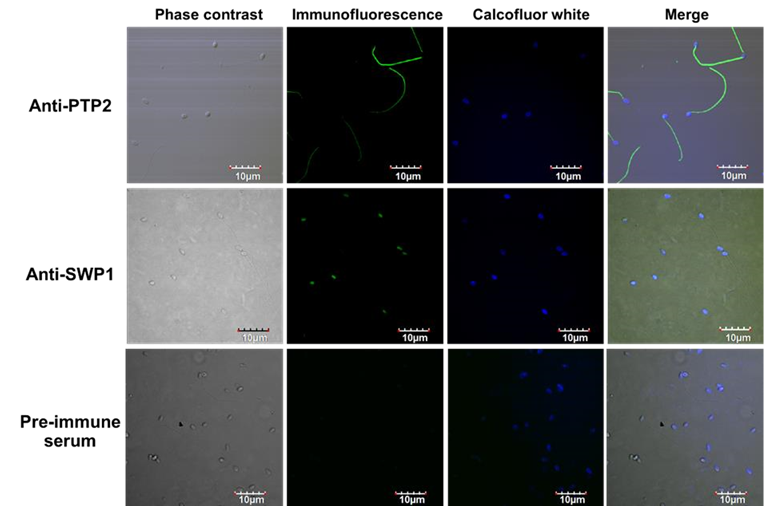


**Figure S2. EhPTP2 localized along extruded polar tubes.** Germinated EHP spores were incubated with an anti-EhPTP2 antibody or an anti-spore wall protein 1 (SWP1) antibody as a primary antibody and with a fluorescence dye-conjugated secondary antibody (Alexafluor-488, Cell Signaling) followed by counter staining with calcofluor white and visualized under a fluorescence microscope. The green signals represent EhPTP2 localization on polar tubes, but not spores. Only SWP1 signals are visible on EHP spore wall.


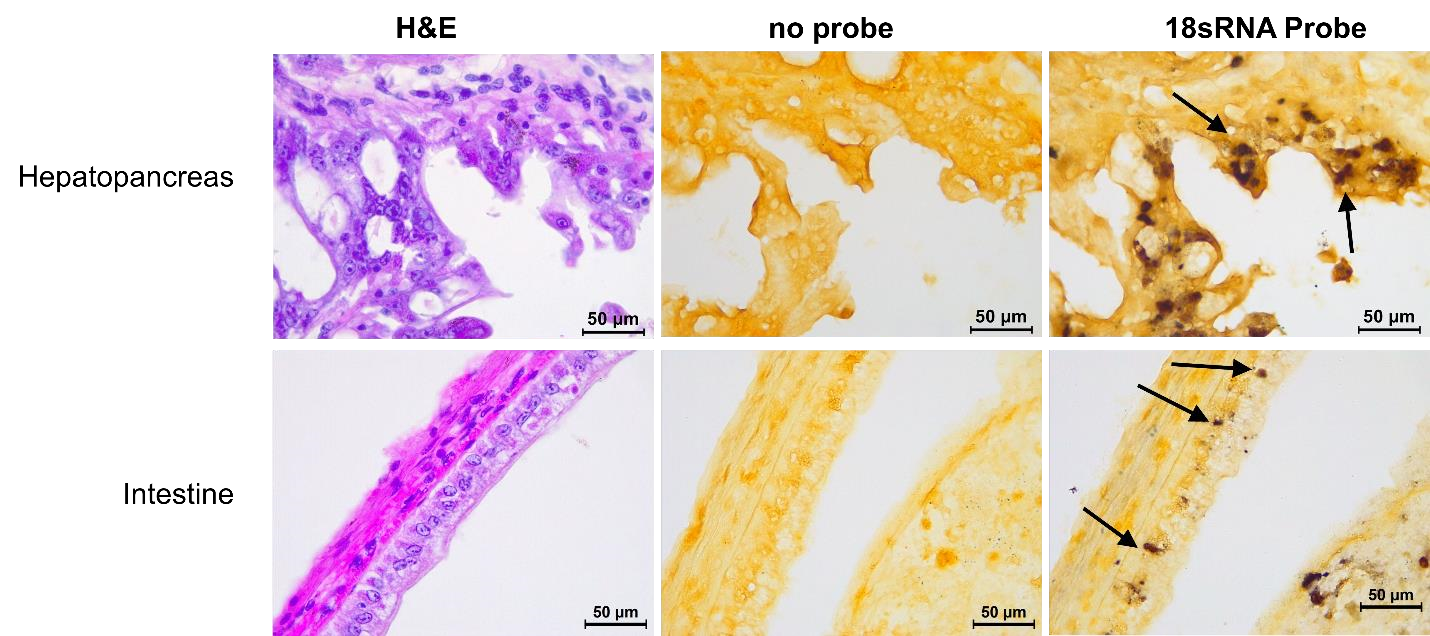


**Figure S3. Detection of EHP by *in situ* hybridization in the shrimp digestive tract post cohabitation.** Naïve shrimp cohabitated with EHP-infected shrimp at day 7 were collected to confirm EHP infection by *in situ* hybridization using an 18sRNA probe. The hepatopancreas and intestine tissue sections were subjected to *in situ* hybridization method following a previous report (Tang et al., 2015). The positive signals of EHP infection (indicated by dark brown color, arrows) were observed in both tissue but not in the stomach which correspond to EhPTP2 detection using the IHC method.


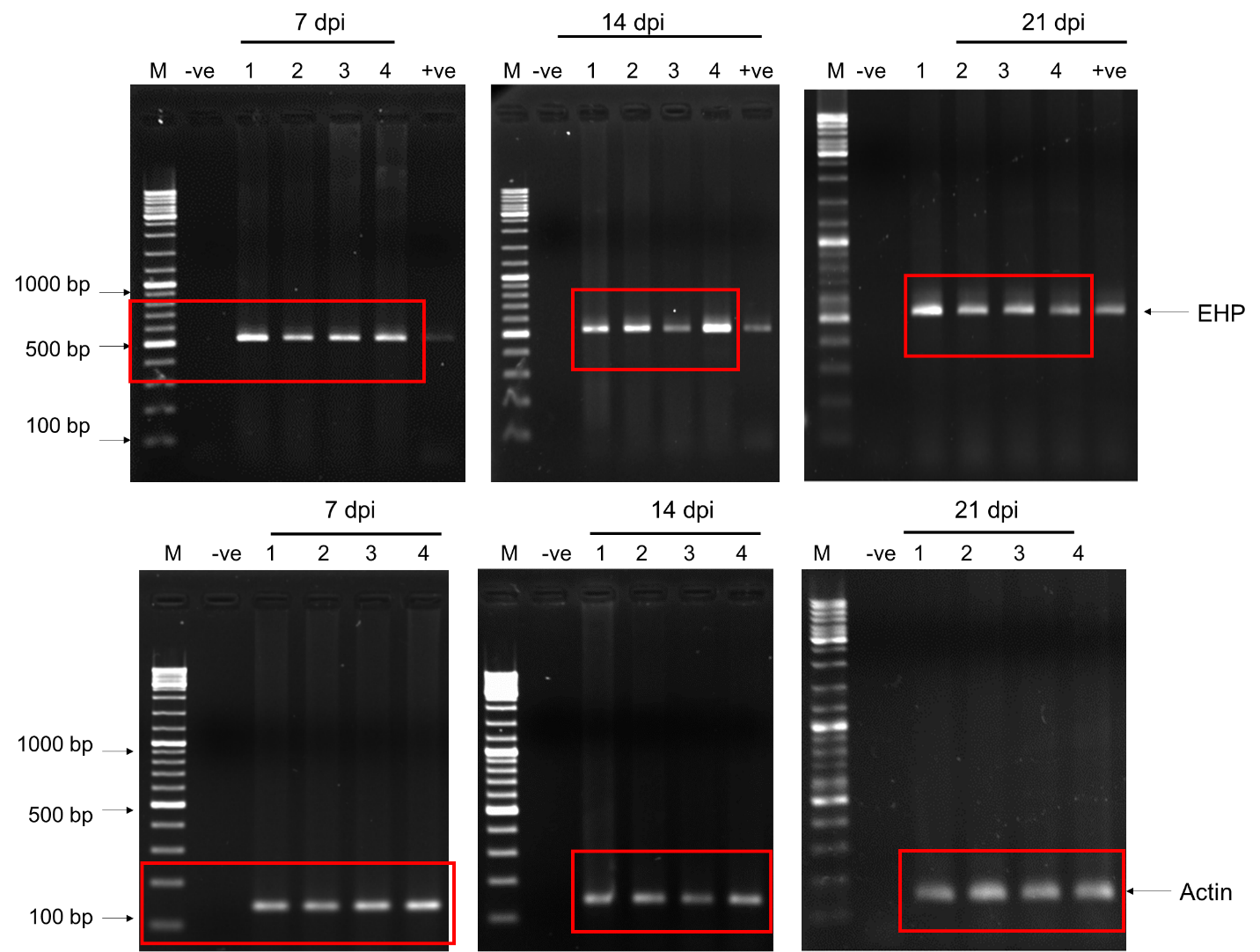


**Figure S4.** Representative agarose gel electrophoresis of EHP detection in shrimp by PCR. Upper panel, the PCR amplicons represent EHP replication based on SWP-PCR detection using SWP primer set. Three sample groups were demonstrated based on time-points post EHP infection under control condition. Lower panel, the amplicons represent internal control gene amplification using actin primer set. The letters “-ve” and “+ve” represent negative (without template) and positive control (plasmid with targeted amplicon inserted) reaction for each sample set of PCR analysis, respectively. Numbers indicate individual shrimp sample. The letter “M” indicates 100 bp-DNA ladder. The images in red boxes were shown in Figure 4B (Control). The image capture and intensities analyses were analyzed by ImageLab software version 6.0 (https://www.bio-rad.com/ImageLab).


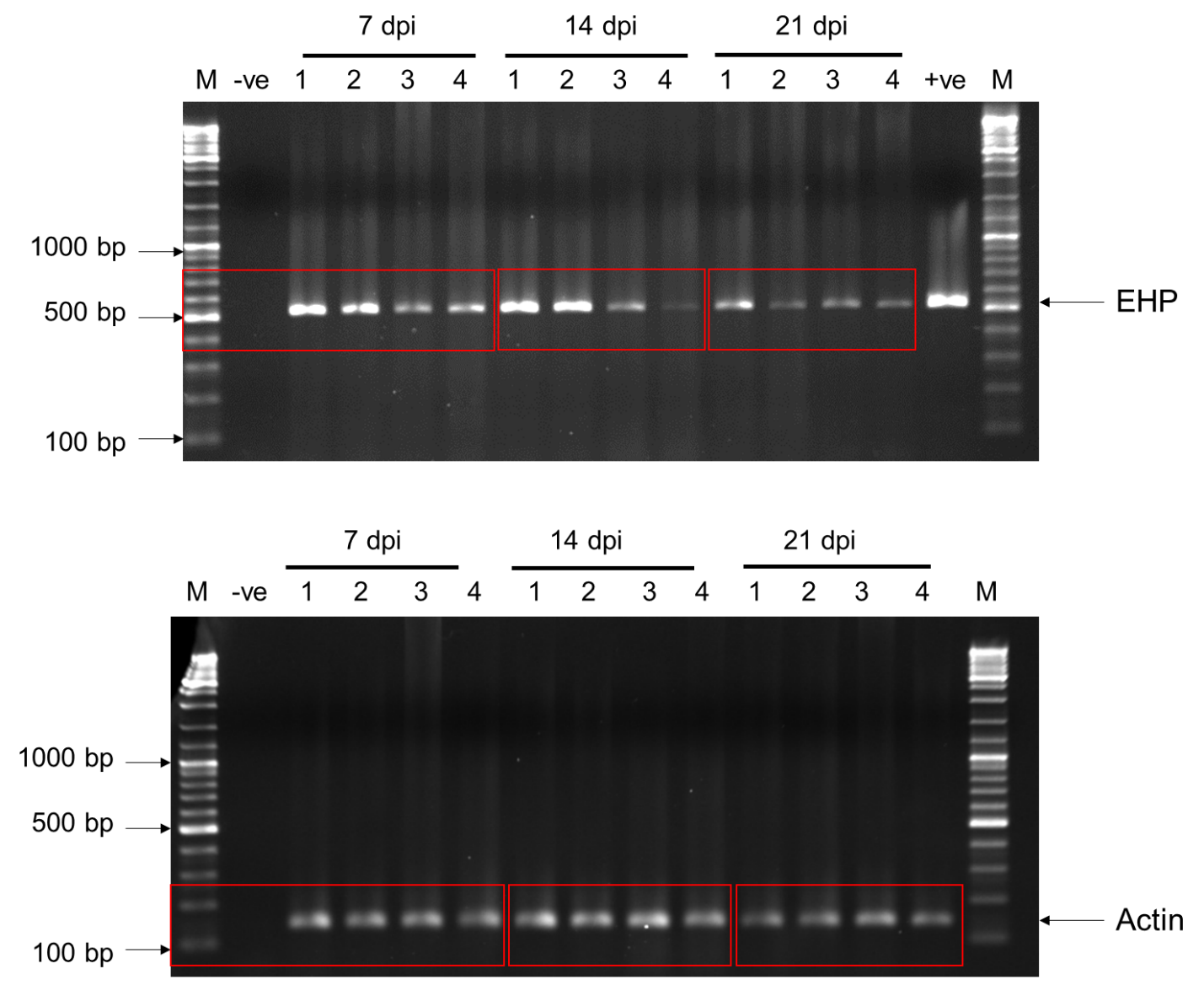


**Figure S5.** Representative agarose gel electrophoresis of EHP detection in shrimp by PCR. Upper panel, the PCR amplicons represent EHP replication based on SWP-PCR detection using SWP primer set. Three sample groups were demonstrated based on time-points post EHP infection under EGFP-dsRNA injection. Lower panel, the amplicons represent internal control gene amplification using actin primer. The letters “-ve” and “+ve” represent negative (without template) and positive control (plasmid with targeted amplicon inserted) reaction for each sample set of PCR analysis, respectively. Numbers indicate individual shrimp sample. The letter “M” indicates 100 bp-DNA ladder. The images in red boxes were shown in Figure 4B (EGFP-dsRNA). The image capture and intensities analyses were analyzed by ImageLab software version 6.0 (https://www.bio-rad.com/ImageLab).


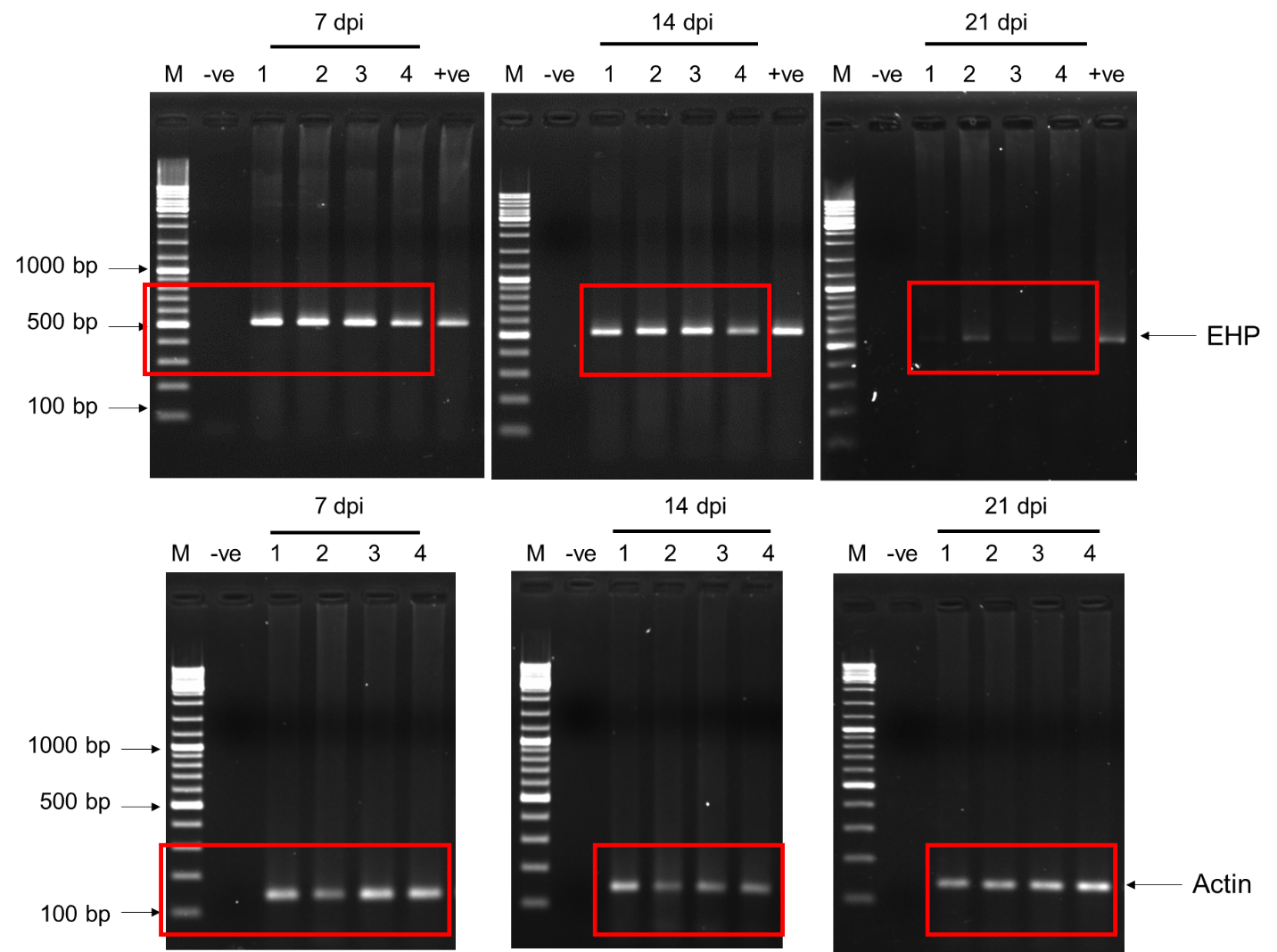


**Figure S6**. Representative agarose gel electrophoresis of EHP detection in shrimp by PCR. Upper panel, the PCR amplicons represent EHP replication based on SWP-PCR detection using SWP primer set. Three sample groups were demonstrated based on time-points post EHP infection under PTP2-dsRNA injection. Lower panel, the amplicons represent internal control gene amplification using actin primer. The letters “-ve” and “+ve” represent negative (without template) and positive control (plasmid with targeted amplicon inserted) reaction for each sample set of PCR analysis, respectively. Numbers indicate individual shrimp sample. The letter “M” indicates 100 bp-DNA ladder. The images in red boxes were shown in Figure 4B (EhPTP2-dsRNA). The image capture and intensities analyses were analyzed by ImageLab software version 6.0 (https://www.bio-rad.com/ImageLab).


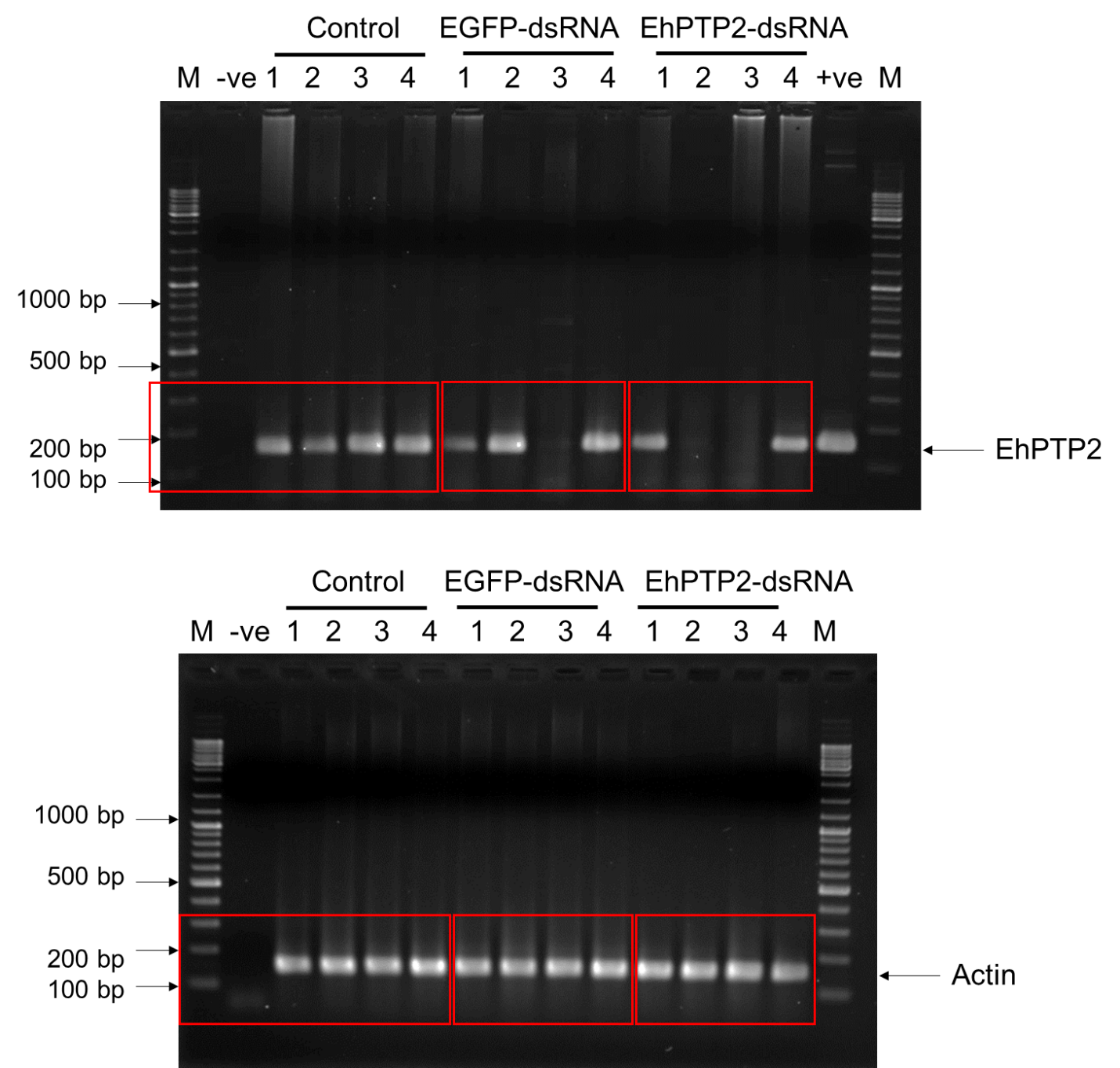


**Figure S7**. Representative agarose gel electrophoresis of EhPTP2 mRNA detection in shrimp by RT-PCR. Upper, PCR gel and amplicons represent EhPTP2 mRNA expression in individual shrimp comparing among three testing conditions. Lower, the gel and amplicons indicate internal control gene detection using actin primer. The letters “-ve” and “+ve” represent negative (without template) and positive control (plasmid with targeted amplicon inserted) reaction for each sample set of PCR analysis, respectively. Numbers indicate individual shrimp sample. The letter “M” indicates 100 bp-DNA ladder. The images in red boxes were shown in Figure 5B (upper panel). The image capture and intensities analyses were analyzed by ImageLab software version 6.0 (https://www.bio-rad.com/ImageLab).


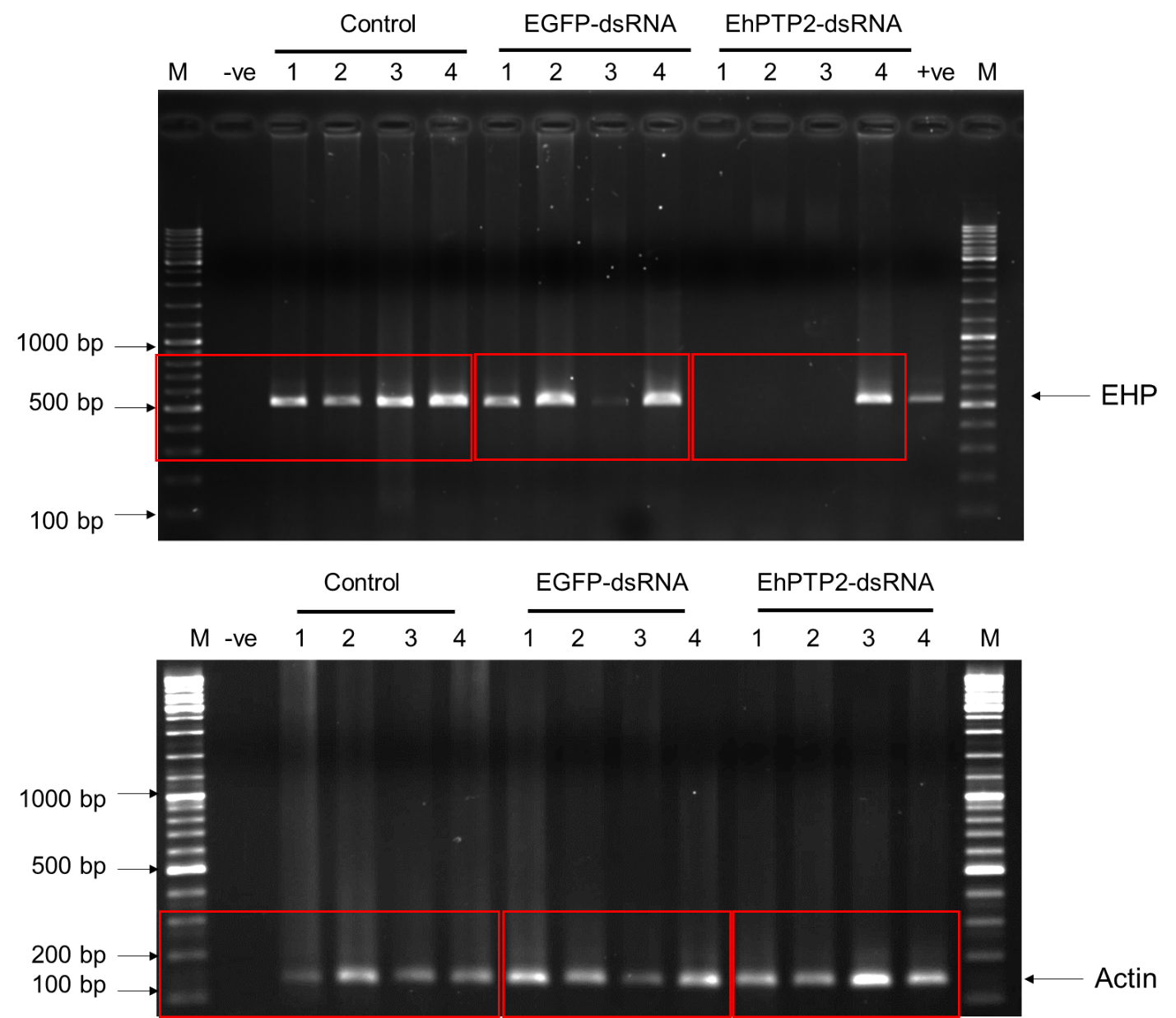


**Figure S8**. Representative agarose gel electrophoresis of EHP detection in shrimp by PCR. Upper panel, PCR gel and amplicons represent EHP replication in individual shrimp comparing among three testing conditions, determined by SWP-PCR primer set. Lower panel, the gel and amplicons indicate internal control gene detection using actin primer. The letters “-ve” and “+ve” represent negative (without template) and positive control (plasmid with targeted amplicon inserted) reaction for each sample set of PCR analysis, respectively. Numbers indicate individual shrimp sample. The letter “M” indicates 100 bp-DNA ladder. The images in red boxes were shown in Figure 5B (lower panel). The image capture and intensities analyses were analyzed by ImageLab software version 6.0 (https://www.bio-rad.com/ImageLab).

**Table S1.** List of primers used in this study.

| Primer name | Sequences (5’-3’) | Purpose | Reference |
| --- | --- | --- | --- |
| SWP-1F | TTGCAGAGTGTTGTTAAGGGTTT | Nested PCR for detection of EHP replication | Jareonlak, et al. 2016 |
| SWP-1R | CACGATGTGTCTTTGCAATTTTC |  |  |
| SWP-2F | TTGGCGGCACAATTCTCAAACA |  |  |
| SWP-2R | GCTGTTTGTCTCCAACTGTATTTGA |  |  |
| PTP2-dsRNAF | TCAAAGCCAATTCAAAAGCC | dsRNA synthesis | This study |
| PTP2-dsRNAR | CCAGATGGTAAGGTAAGGTGGTTGG |  |  |
| PTP2-dsRNAT7F | taatacgactcactatagggTCAAAGCCAATTCAAAAGCC |  |  |
| PTP2-dsRNAT7R | taatacgactcactatagggCCAGATGGTAAGGTAAGGTGGTTGG |  |  |
| PTP2-F | CGTAAACAGGGCAGGAGATATG | RT-PCR for PTP2 transcript detection | This study |
| PTP2R | CATTGGATCGCATGTGTTCTTG |  |  |
| Pv-actin_F | TTCCGACTCCAAGAACGACC | Internal control gene amplification | This study |
| Pv-actin-R | GAGCAGTGTGGCAATCAAGC |  |  |
